# Icaritin promotes apoptosis and inhibits proliferation by down-regulating AFP gene expression in hepatocellular carcinoma

**DOI:** 10.1186/s12885-021-08043-9

**Published:** 2021-03-25

**Authors:** Hui Li, Yujuan Liu, Wei Jiang, Junhui Xue, Yuning Cheng, Jiyin Wang, Ruixiang Yang, Xiaowei Zhang

**Affiliations:** Department of Biochemistry and Biophysics, School of Basic Medical Sciences, Beijing Key Laboratory of Protein Posttranslational Modifications and Cell Function, Peking University Health Science Center, 100191 Beijing, People’s Republic of China

**Keywords:** Icaritin, AFP, Hepatocellular carcinoma, Hepatocellular proliferation, p53 ubiquitination

## Abstract

**Background:**

Icaritin, an active ingredient of the Chinese herb Epimedium, plays an anti-tumor role in liver cancer by inhibiting the proliferation of hepatocellular cells and promoting their apoptosis. In China, phase II and a large phase III clinical trial of icaritin reagent for the treatment of hepatocellular cancer is under-going, but the specific mechanism of icaritin action was unclear. Alpha-fetoprotein (AFP), an oncofetal protein, produced in the healthy fetal liver and yolk sac. Intracellular AFP promoted cellular proliferation and inhibited cellular apoptosis in hepatocellular carcinoma (HCC). The study was aimed to investigate the effect of icaritin on HCC through p53/AFP pathway.

**Methods:**

Real-time RT PCR and western blot were used to detect p53 and AFP expression levels in HCC cells treated with icaritin. The mechanism of icaritin affecting p53 expression was verified by ubiquitination experiment, and the binding activity of icaritin on p53 in AFP promoter region was verified by luciferase experiment. EdU, MTT and flow cytometry were used to determine whether icaritin affected HCC cellular proliferation and apoptosis through p53/ AFP pathway. Expression levels of p53 and AFP in xenograft mouse model were determined by western blotting.

**Results:**

Our results showed icaritin inhibited AFP expression at mRNA and protein level. AFP was also identified as the target gene of the p53 transcription factor. Icaritin abrogated murine double minute (Mdm) 2-mediated p53 ubiquitination degradation to improve the stability of p53. Up-regulated p53 protein levels then transcriptionally inhibited the AFP promoter. Icaritin-mediated decrease of AFP through Mdm2/p53 pathways inhibited HCC cellular proliferation and promoted HCC cellular apoptosis.

**Conclusion:**

Our findings revealed the mechanism of icaritin in promoting apoptosis and inhibiting proliferation in liver cancer cells. The regulatory mechanism of icaritin in AFP protein down-regulation provides a theoretical and experimental basis for further research into new drugs for the treatment of liver cancer.

**Supplementary Information:**

The online version contains supplementary material available at 10.1186/s12885-021-08043-9.

## Background

Alpha-fetoprotein (AFP) is an oncofetal protein produced in the healthy fetal liver and yolk sac. It is undetectable or rarely detected in adults. However, AFP expression is up-regulated in 70–80% of patients with hepatocellular carcinoma (HCC), and is a known tumor marker in the clinical diagnosis of HCC [1, 2]. Clinical studies showed that high expression of serum AFP was closely associated with a high degree of HCC malignancy [3].

In recent years, an increasing number of studies have focused on the role of intracellular AFP in promoting cell growth and inhibiting cell apoptosis [4]. We previously demonstrated that intracellular AFP promoted cell proliferation through binding with caspase 3 to block caspase 8 apoptosis signal transmission, and binding with phosphatase and tensin homolog to relieve inhibition of the phosphoinositide-3-kinase/AKT pathway. We also confirmed that cytoplasmic AFP blocked retinoic acid/retinoic acid receptor-mediated expression of *GADD153*, *GADD45A*, and *Fn14* and that down-regulation of these genes led to the abnormal growth of HCC cells [5–8]. These results showed the importance of serum or cytoplasmic AFP in promoting cellular proliferation and inhibiting cellular apoptosis in HCC. Therefore, the down-regulation of circulating or cytoplasmic AFP expression may be helpful in the treatment of liver cancer. Indeed, in recent years, AFP has been used as an immunotherapy target for HCC [9].

Icaritin is an active ingredient of the Chinese herb *Epimedium*. It has a wide range of biological and pharmacological functions, including antioxidative, anticancer, and enhancing immunity [10–12]. Its anticancer activities were reported in breast cancer, lung cancer, esophageal cancer, glioblastoma, leukemia, and HCC [13–17]. Previous studies found that icaritin inhibited the growth of liver cancer cells by promoting HCC cell apoptosis through activating the caspase pathway and inhibiting the interleukin-6/Janus kinase (JNK)2/signal transducer and activator of transcription 3 signaling pathway, while the safety of daily doses of oral icaritin (1600 mg) was documented in clinical studies [18, 19]. Icaritin has been shown to exert a therapeutic effect in HCC, and a clinical trial involving icaritin treatment of HCC [NCT013236636] has entered its third phase [20]. However, the specific anticancer mechanisms of icaritin remain to be clarified. Considering the importance of AFP in the development of liver cancer, we considered whether icaritin inhibits the proliferation of liver cancer cells by down-regulating AFP protein expression.

We recently demonstrated that icaritin inhibited the expression of cytoplasmic AFP in hepatitis B virus-infected hepatoma cells [21]. However, the intrinsic mechanism of this was unknown. Therefore, the present study aimed to determine whether icaritin induces HCC cell apoptosis by inhibiting cytoplasmic AFP expression, and how this is done. Clarification of these mechanisms will provide a theoretical and experimental basis for further research into the use of icaritin in liver cancer treatment.

## Methods

### Reagents

Icaritin with a purity of up to 99% was a gift from Dr. Kun Meng (Shenogen Biomedical Co., Ltd.) A stock solution was dissolved in dimethyl sulfoxide (DMSO) at various concentrations (2.5, 5, 10, 20, and 40 mM) and stored at − 20 °C.

### Cell culture

The human HepG2 cell line (AFP-positive and p53-wild-type HCC cell line) was obtained from China Infrastructures of Cell Line Resource and SMMC7721 cell line (AFP-positive and p53-wild-type HCC cell line) was provided by Prof. Fengmin Lu [22] (Peking University Health Science Centre, China). HepG2 and SMMC7721 cells separately maintained in high glucose Dulbecco’s modified Eagle medium (Invitrogen) supplemented with 10% fetal bovine serum (FBS) and RPMI 1640 medium (Invitrogen) supplemented with 10% FBS, respectively. PLC cells (AFP-positive and p53-mutant-type HCC cell line) and L02 cells (a normal human liver cell line that produces no detectable AFP) were provided by Prof. Fengmin Lu [8] (Peking University Health Science Centre, China). and maintained in high glucose Dulbecco’s modified Eagle medium (Invitrogen) supplemented with 10% fetal bovine serum (FBS, Gibco). Cells were grown at 37 °C in 5% CO_2_. Medium was changed to phenol red-free medium with 10% FBS before icaritin was added. All cells were confirmed to be negative for mycoplasma by Mycoplasma Detection Kit (Solarbio, Beijing, China).

### Plasmids and transfection and lentivirus gene expression

Pluc-1 contains 1.8 kb of DNA from the human AFP gene upstream of the translational start site. Luc1–700mut/luc1-900mut contained a mutated p53 site and luc1–700/900mut contained two mutated p53 sites. Mutated primer DNA sequences are listed as follows:

- Luc1–700mut 5′-CACTTTATAAAGACAAGCGTGCAAATAAAATT-3′ and
- 5′-GCTTGTCTTTATAAAGTGGTCAGGTGCATC − 3′
- Luc1-900mut 5′-GGTCTGGGTTACAGAAGCGGCATTGGGAAT-3′ and
- 5′-GCTTCTGTAACCCAGACCAGTTAAATCAGAAT-3′

Cells were transiently transfected with plasmids using Lipofectamine 2000 reagent (Invitrogen, Waltham, MA, USA) following the manufacturer’s protocol. At 28 h after transfection, cells were treated with icaritin for 20 h, then harvested and lysed for the detection of *AFP* promoter activity. The lentivirus vector pLL3.7-shp53 expresses short hairpin (sh) RNA targeting p53 mRNA (5′-CCACTTGAUGGAGAGTATT-3′) as previously described [23]. The vector was used to create p53 knockdown cells.

### Western blot and antibodies

Western blot was performed for the analysis of AFP and p53 expression in HCC cell lines. Briefly, cells were lysed in lysis buffer, and 30 μg of protein was utilized for each western blot. Primary antibodies were against AFP (145501–1-AP, Proteintech), p53 (sc-126, Santa Cruz Biotechnology), Mdm2 (sc-965, Santa Cruz Biotechnology), Flag (F1804, Sigma-Aldrich), p-p53(sc-377,561, Santa Cruz Biotechnology), Arf^p14^(sc-53,639, Santa Cruz Biotechnology), pro-caspase-3 and cleaved-caspase-3 (14,220, CST), PTEN (9188, CST) and GAPDH (KM9002, Sungene Biotech). The secondary antibodies IRDye 800-conjugated anti-mouse IgG antibody (610–132-121) and DyLight 800-conjugated affinity-purified anti-rabbit IgG (611–145-002) were purchased from Rockland. Immunocomplexes were visualized by Odyssey infrared imaging system (LI-COR Bioscience, Lincoln, NE).

### Real-time quantitative -PCR

Total RNA was isolated using the RNAsimple Total RNA kit (Tiangen). The cDNA was synthesized using ReverAid First Strand cDNA Synthesis kit (Thermo Scientific) and then analyzed by real-time PCR analysis with Maxima SYBR Green qPCR Master Mix (Thermo Scientific). The relative content of *AFP* and p53 mRNA was presented as a fold-change compared with the control. Primer DNA sequences are listed as follows:

- Human p53: 5′-TAACAGTTCCTGCATGGGCGGC-3′ and
- 5′-AGGACAGGCACAAACATGCACC-3′
- Human AFP: 5′-CCAACAGGAGGCCATGCTT-3′ and
- 5′-GAATGCAGGAGGGACATATGTTT-3′

### Co-immunoprecipitation (CoIP)

Cells for the immunoprecipitation assay were lysed in IP lysis buffer (25 mM Tris-HCl pH 7.4, 150 mM NaCl, 1% NP-40, 1 mM EDTA, 5% glycerol) containing Protease Inhibitor Cocktail (Sigma). Cell extracts were incubated with specified antibodies or control IgG overnight at 4 °C with constant rotation. Protein A Sepharose (GE) was then added to the complexes for 2 h, which were washed three times with IP lysis buffer, and subsequently resolved by sodium dodecyl sulfate (SDS)–polyacrylamide gel electrophoresis (PAGE) followed by western blotting analysis.

### Chromatin immunoprecipitation (ChIP)

A total of 2 μg of normal IgG or antibodies against p53 was used to perform the ChIP assay using Chromatin Immunoprecipitation (ChIP) Assay Kit (Millipore) following ChIP assay instructions. PCR was used to detect binding between p53 and the *AFP* promoter*.* Quantitative (q)PCR was performed to determine the influence of icaritin on the capacity of p53 binding to DNA. Data processing was achieved according to our previously published articles [23]. Primer DNA sequences are listed as follows:

- p53_700_: 5′-TGAGCCACTCTTAGCATCCA-3′ and
- 5′-GCACAAGCCCTAATAAACCAAGT-3′
- p53_900_: 5′-GGGTCTCAACTCCACAGATT-3′ and
- 5′-CCCGATCTTGGCTACACATT-3′

### Luciferase reporter assay

Luciferase reporter assays were performed. HepG2 and SMMC7721 cells were transfected with plasmids including the *AFP* promoter region using Lipofectamine 2000 reagent and then treated with 20 μM icaritin for 20 h. Then, cells were harvested and lysed with reagents from the dual luciferase reporter assay kit (Promega) for the detection of transfection efficiency. Firefly luciferase activity measurements were normalized to Renilla luciferase activity. The original pGL3-basic vector served as a negative control.

### In vivo ubiquitination assay

To detect p53 ubiquitination levels, HepG2 and SMMC7721 cells were cotransfected with various plasmids or treated with 20 μM icaritin. A total of 14 h after transfection and icaritin treatment, cells were treated with 10 μM MG132 (Merck Millipore) for 6 h, then whole cell lysates prepared by Flag-lysis buffer (50 mM Tris-HCl pH 7.8, 137 mM NaCl, 10 mM NaF, 1 mM EDTA, 1% Triton X-100, 0.2% Sarkosyl, 1 mM DTT, 10% glycerol, and fresh protease inhibitors) were immunoprecipated with an anti-p53 antibody and resolved by SDS–PAGE followed by western blot analysis.

### Protein half-life assay

The protein half-life assay was used to detect the influence of icaritin on p53 post-transcriptional regulation. HepG2 and SMMC7721 cells were treated with icaritin as indicated in individual experiments. A total of 20 h after treatment, 100 μg/ml of cycloheximide was added to the dishes, and this treatment was terminated after 0, 15, 30, 45, 60, or 120 min as indicated. Whole cell lysates were collected and 20 μg or 40 μg of total protein from each sample was analyzed by western blot with an anti-p53 antibody. P53 protein quantification was determined using IMAGE-J software, normalized to GAPDH.

### Flow cytometric analysis for apoptosis

Flow cytometry was performed to determine the effect of icaritin and AFP on apoptosis. After treatment of HepG2 cells (+/− p53) and SMMC7721 cells (+/− p53) for 24 h with icaritin, apoptosis induced by icaritin was analyzed by flow cytometry. Cells were collected and re-suspended in 70% ethanol after washing. They were thenstained using an Annexin V/propidium iodide (PI) Apoptosis Detection Kit (Dojindo Laboratories, Kumamoto, Japan) following the manufacturer’s instructions. Relative fluorescent intensities of PI staining were measured using a FACScan-420 flow cytometer (Becton Dickinson). The extent of cellular apoptosis was determined according to DNA analysis. This experiment was repeated at least three times.

### MTT assay

HepG2 and SMMC7721 cells were stably transfected with either control plasmid or vector pLL3.7-shp53 following selection with puromycin (0.4 μg/ml) and/or G418 (400 μg/ml), cells were seeded into 96-well plates at a density of 2000 cells/well. After culturing for 2, 4, 6, 8, 12, 20, or 24 h, 15 μl of MTT solution (5 mg/ml) was added to each well, followed by further incubation at 37 °C for 4 h. Medium was then removed and 200 μl of DMSO was added to each well to dissolve the formazan crystals. Absorbance at 490 nm was determined using a microplate reader.

### EdU proliferation assay

DNA synthesis in HepG2 and SMMC7721 cells was performed by the EdU incorporation assay (RIBOBIO) following the manufacturer’s instructions. Cells were incubated with an EdU-labeling solution for 2 h at 37 °C and fixed with 4% paraformaldehyde for 30 min. After permeabilization, cells were reacted with the reaction solution for 30 min. Subsequently, cell nuclei were stained with 1 × Hoechst 33342 for 30 min, then photographed under a fluorescent microscope (Olympus). Finally, the proliferation rate was calculated.

### Tumorigenicity in nude mice

The experimental animal facility has been accredited by the AAALAC (Association for Assessment and Accreditation of Laboratory Animal Care International). Four-week-old male BALB/c nude mice (*N* = 8, average weight 20 ± 2 g) were purchased from Department of Laboratory Animal Science in Peking University Health Science Center. All mouse experiments conformed to the Guide for the Care and Use of Laboratory Animals of the Health Science Center of Peking University. Mice were housed in groups with 12-h dark-light cycles and had free access to food and water. Mice were sacrificed under the isoflurane inhalation and followed by cervical dislocation.

### Statistical analysis

The results of multiple observations are presented as the mean ± standard deviation of at least three separate experiments. Statistical significance was determined using the Student’s t test (SPSS 17.0 software). *P* values *p* < 0.01 or 0.05 were considered statistically significant.

## Results

### Icaritin inhibited AFP at the transcription level in hepatoma cells

**Fig. 1:**
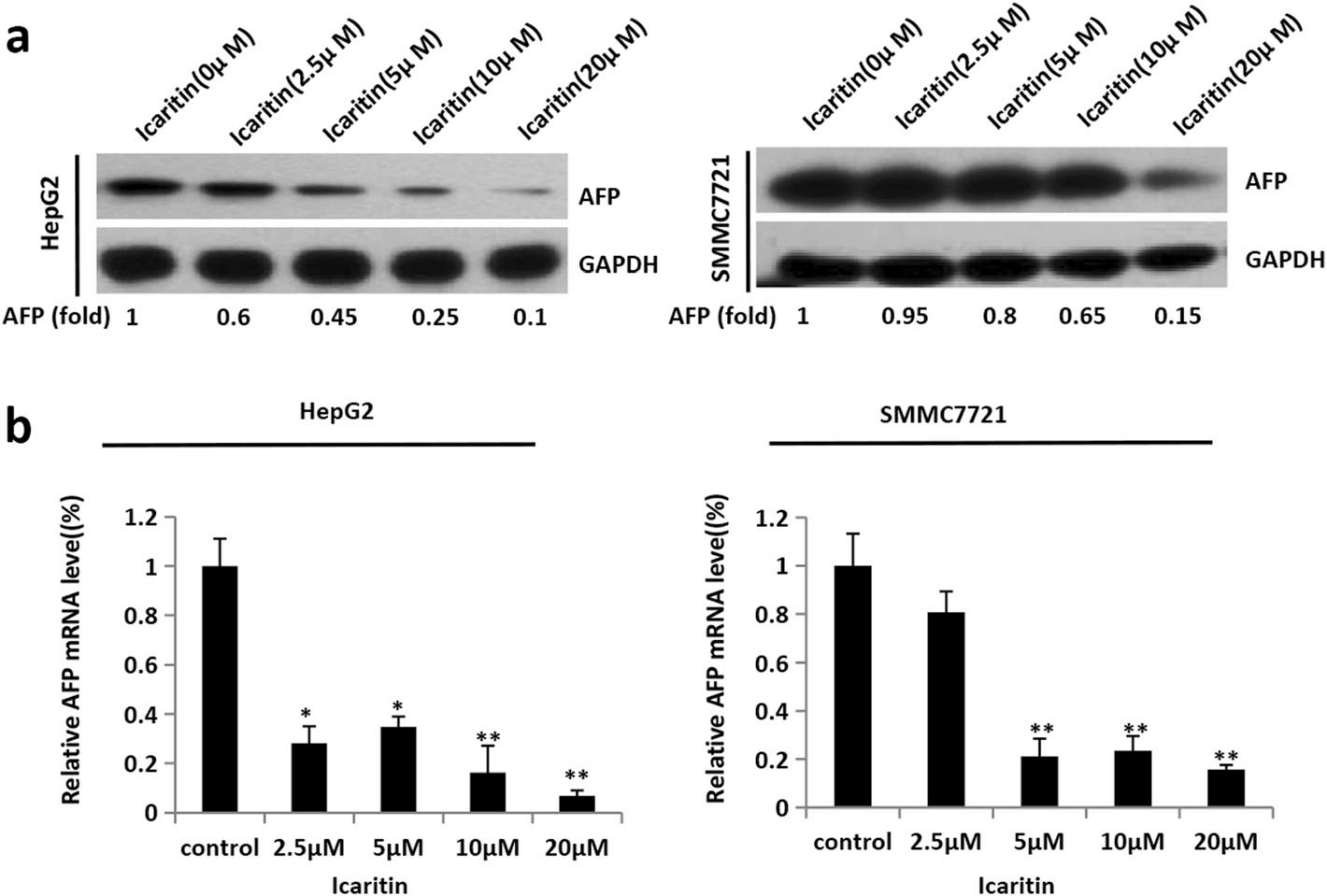
Icaritin inhibited AFP at the transcription level in hepatoma cells. The expression of AFP gene was detected in HepG2 and SMMC7721 cells treated with icaritin at different concentrations. **a** Icaritin inhibited AFP expression at protein level. The protein expression of AFP and GAPDH (as a control) was measured by western blot in HepG2 and SMMC7721 cells treated with icaritin at different concentrations for 20 h. **b** The mRNA expression of AFP was measured by qRT-PCR in HepG2 and SMMC7721 cells treated with icaritin. Results are representative of three independent experiments, and values are the mean ± S.E. *, *p* < 0.05; **, *p* < 0.01. The full-length images for blots in Fig. 1**a** were presented in Supplementary Fig. 4

Cytoplasmic AFP has been shown to promote tumor cell proliferation, inhibit cell apoptosis, and to play an important role in HCC occurrence and development. To determine the effect of icaritin on AFP expression, western blotting and qRT-PCR were used in icaritin-treated and -untreated hepatoma cells (HepG2 cells and SMMC7721 cells). As shown in Fig. 1, icaritin inhibited AFP expression at both the mRNA and protein level. AFP protein expression gradually decreased with increasing doses of icaritin in HepG2 cells and SMMC7721 cells (Fig. 1a), becoming most apparent after icaritin treatment for 20 h at a dose of 20 μM. qRT-PCR showed that *AFP* mRNA was down-regulated after icaritin treatment for 20 h at a dose of 20 μM in HepG2 and SMMC7721 cells (Fig. 1b). These results indicated that icaritin inhibited *AFP* expression at the transcriptional level.

### Icaritin inhibited AFP expression by increasing p53 protein expression

**Fig. 2:**
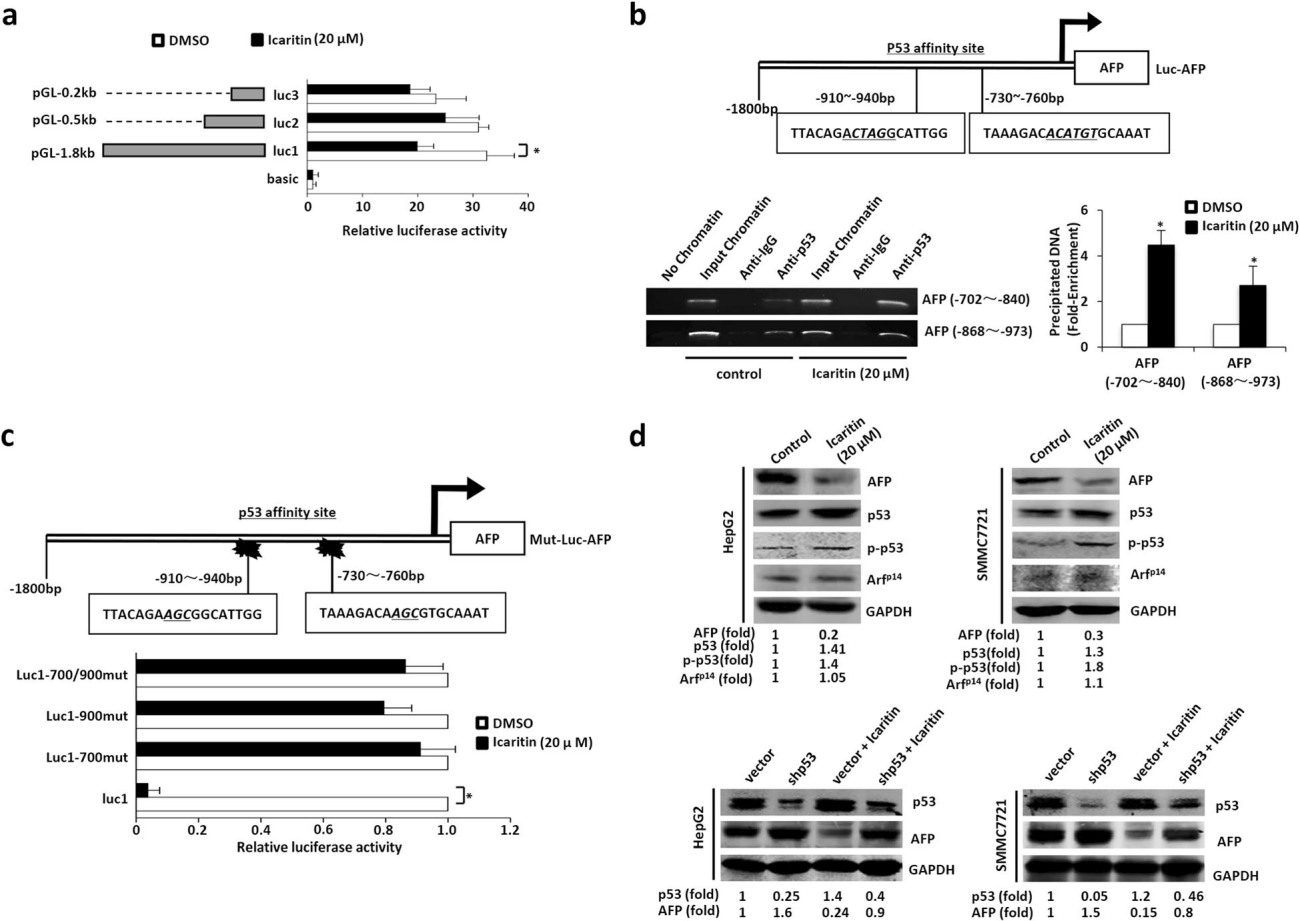
Icaritin inhibited AFP expression by increasing p53 protein expression. **a** Icaritin inhibits the activity of AFP promoter. Three AFP-Luc fusion constructs (pLuc1–3) with different length fragments (1871, 448 and 215 bp) were transfected into HepG2 cells following 20 μM icaritin treatment. The luciferase activities were expressed as mean ± S.E. *, *p* < 0.05. **b** Schematic diagram of the AFP promoter. Shown is the p53 affinity site within the AFP promoter at positions − 910 to − 940 and − 730 to − 760 from the translational start site (Luc-AFP, top). ChIP assay was performed to detect p53 affinity in AFP 5′ promoter region and the effect of icaritin on it. The regions from position − 702 to position − 840 and from position − 868 to − 973 were analyzed by specific PCR. Anti-p53 antibody was used in the indicated lanes. ChIP realtime PCR was performed to identify the effect of icaritin on p53 affinity. **c** Mut-Luc1-AFP is a mutant AFP promoter with a mutation in the p53 affinity site (bottom). Three p53-binding defective mutants (luc1–700mut, luc1-900mut or luc1–700/900mut) were transfected into HepG2 cells following icaritin treatment. The luciferase activities were expressed as mean ± S.E. **d** Icaritin decreased AFP expression and increased p53 expression. AFP, p53, p-p53, Arf^p14^ and GAPDH (as a control) protein were detected by western blot in HepG2 and SMMC7721 cells after DMSO or icaritin treatment (top). P53, AFP and GAPDH (as a control) protein were detected by western blot in HepG2 cells and SMMC7721 cells with vector, shp53, vector+icaritin or shp53 + icaritin (bottom). Results are representative of three independent experiments, and values are the mean ± S.E. *, *p* < 0.05; **, *p* < 0.01. The full-length images for gels/blots in Fig. 2**b** and **d** were presented in Supplementary Fig. 5

To analyze the activity of elements in the 5′ regulatory region of *AFP* and the impact of icaritin on the *AFP* promoter, we constructed three *AFP* promoter–luciferase reporters with the native *AFP* segment (1871 bp, 448 bp, or 215 bp from the translational start site), designated pLuc1, pLuc2, and pLuc3, respectively. The three plasmids were transfected into HepG2 cells following 20 μM icaritin treatment. Analysis with the luciferase activity assay showed that icaritin repressed the pLuc1 promoter compared with the other two constructs (Fig. 2a), possibly by acting as a silencer.

P53-mediated repression of *AFP* expression has been reported to involve direct interaction with a p53 DNA binding element or chromatin structure alteration at the core promoter [24]. Therefore, pLuc-1 was analyzed and predicted to contain two new p53 affinity sites at − 730 to − 760 bp and − 910 to − 940 bp in the 5′ regulatory region of *AFP* (Fig. 2b). The ChIP assay was used to validate the p53 affinity sites. The result of ChIP assay showed icaritin enhanced the ability of p53 binding to the promoter of *AFP* in vivo (Fig. 2b). Three p53-binding defective mutants (luc1–700mut, luc1-900mut, and luc1–700/900mut) containing one or two point mutations in the p53 binding element that abolish p53 binding were used to investigate sequence-specific repression of the *AFP* promoter. Icaritin treatment decreased native *AFP* promoter activity, but had no effect on that of p53-binding defective mutants (Fig. 2c). Western blot demonstrated that p53 protein levels and phosphorylated p53 were up-regulated and AFP was down-regulated after 20 μM icaritin treatment for 20 h in HepG2 and SMMC7721 cells (Fig. 2d). While icaritin had no effect on the expression of p53 positive regulator ARF^p14^. This indicated Arf^p14^ was not involved in the up-regulation of p53 induced by icaritin. When p53 protein was knocked-down, the expression of AFP was increased. Moreover, icaritin up-regulated p53 expression and suppressed AFP expression compared with p53 knockdown group (Fig. 2d). These results further demonstrated that p53 negatively regulated AFP expression through direct binding to the *AFP* promoter, and that icaritin down-regulated *AFP* expression through up-regulating p53 protein expression.

### Icaritin up-regulated p53 protein at the post-transcriptional level

**Fig. 3:**
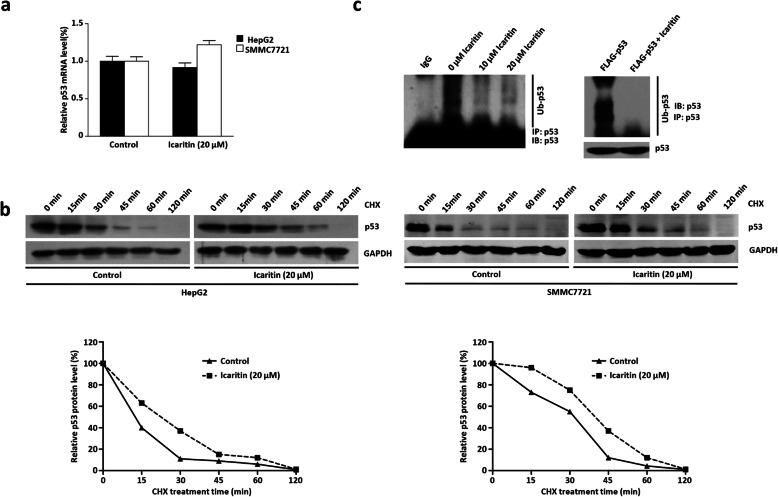
Icaritin up-regulated p53 protein at the post-transcriptional level (**a**) Icaritin had no effect on p53 mRNA expression. P53 gene expression was detected by qRT-PCR after 20 μM icaritin treatment in HepG2 cells and SMMC7721 cells. **b** Icaritin prolongs the half-life of p53. HepG2 cells and SMMC7721 cells were treated with icaritin or Control DMSO respectively for 20 h. Cells were incubated with the protein translation inhibitor cycloheximide (CHX) for 0, 15, 30, 45, 60, or 120 min before harvest. P53 and GAPDH (as a control) protein levels were detected by western blot. **c** Icaritin inhibited p53 ubiquitination in vivo. HepG2 cells were treated with DMSO or 10 μM icaritin or 20 μM icaritin for 14 h and then exposed to 10 μM MG132 for another 6 h prior to lysis. IP assay was carried out by using anti-p53 antibody and followed by western blot to detect Ub-p53 with p53 antibody (left). HepG2 cells were transfected with FLAG-p53 following DMSO or icaritin treatment. IP assay was carried out by using p53 antibody and followed by western blot to detect Ub-p53 with p53 antibody (upper right). The same samples were immunoblotted against p53 to determine immunoprecipitation efficiency (lower right). The full-length images for blots in Fig. 3**b** and **c** were presented in Supplementary Fig. 6

The above results indicated that p53 protein expression was increased after icaritin treatment. We used qRT-PCR to determine whether icaritin affected p53 expression at the transcriptional level. As shown in Fig. 3a, icaritin treatment had no effect on p53 mRNA in HepG2 and SMMC7721 cells, suggesting that p53 protein may be regulated by icaritin at the post-transcriptional level. Therefore, we investigated p53 protein stability using a half-life assay. As shown in Fig. 3b, the half-life of p53 was prolonged after icaritin treatment. We next sought to elucidate the potential mechanism by which icaritin enhances p53 protein stability using a p53 ubiquitination assay. HepG2 cells were harvested 14 h after the addition of icaritin following treatment with 10 μM proteasome inhibitor MG132 for 6 h, and ubiquitinated p53 was detected by western blotting. As shown in Fig. 3c, icaritin inhibited both endogenous p53 ubiquitination degradation (left panel) and the ubiquitination degradation of overexpressed exogenous p53 (right panel). Together, these results suggest that icaritin enhanced p53 stability by inhibiting p53 ubiquitination and degradation.

### Icaritin stabilized p53 by inhibiting Mdm2-mediated p53 ubiquitination

**Fig. 4:**
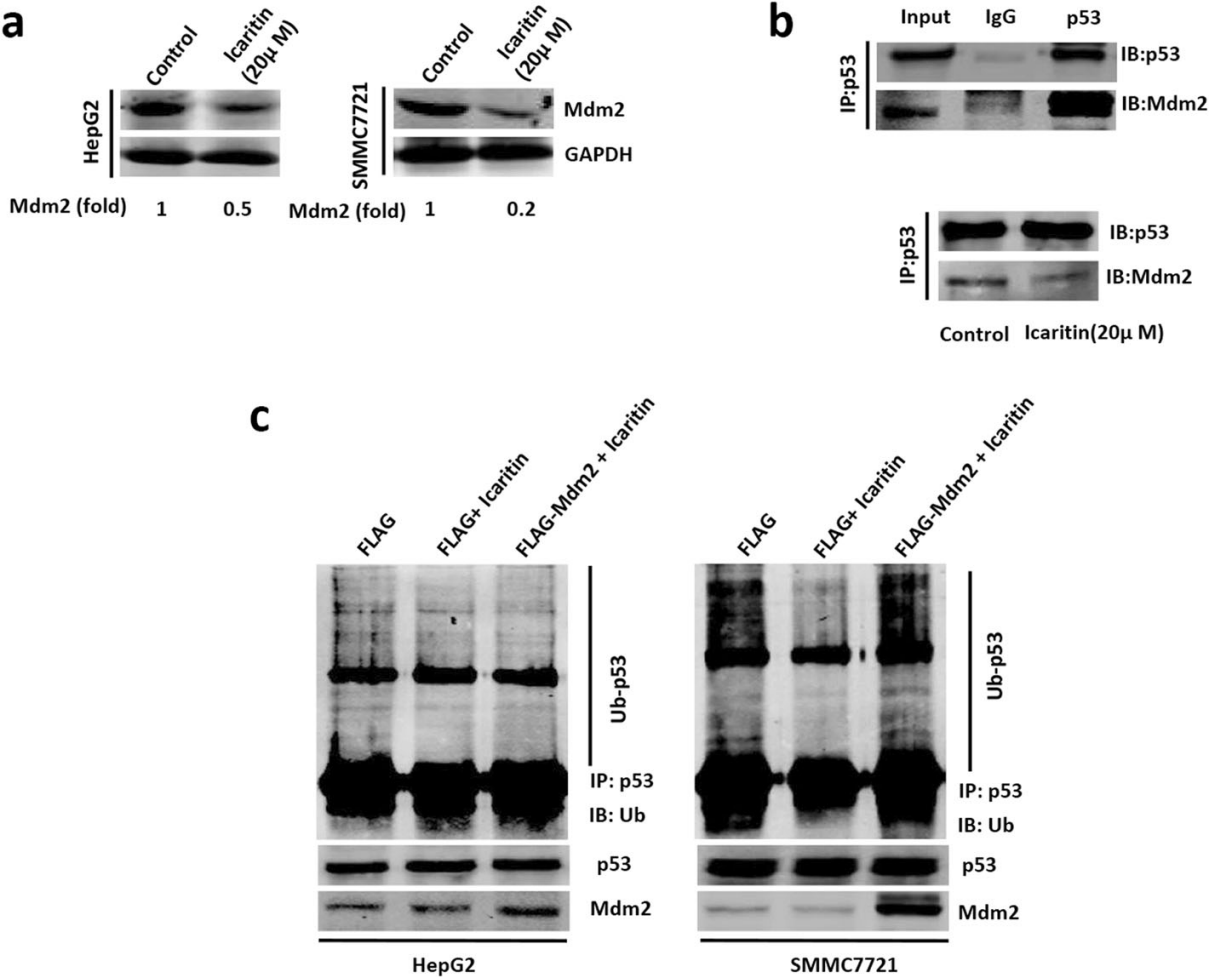
Icaritin stabilized p53 by inhibiting Mdm2-mediated p53 ubiquitination. **a** Icaritin decreased expression of Mdm2. Mdm2 and GAPDH (as a control) was detected by western blot after DMSO or 20 μM icaritin treatment in HepG2 cells and SMMC7721 cells. **b** Icaritin inhibited interaction of protein Mdm2 and p53. IP assay was carried out by using p53 antibody and followed by western blot with p53 or Mdm2 antibody (top). After DMSO or icaritin treatment, IP assay was carried out by using p53 antibody and followed by western blot with p53 or Mdm2 antibody. Samples were immunoblotted against p53 to determine immunoprecipitation efficiency (lower right). **c** Overexpression of Mdm2 rescued p53 ubiquitination in HepG2 and SMMC7721 cells. HepG2 and SMMC7721 cells were cotransfected with vector FLAG, FLAG-Mdm2, HA-ubiquitin and then DMSO or icaritin treatment. P53 protein was then isolated by immunoprecipation and Ub-p53 was detected by western blot using anti-ub antibody. P53 and Mdm2 were detected by western blot in cells cotransfected with FLAG, FLAG+icaritin, FLAG-Mdm2 + icaritin. The full-length images for gels/blots in Fig. 4**a**, **b** and **c** were presented in Supplementary Fig. 7

The E3 ubiquitin ligase Mdm2 is a major negative regulator of p53 expression, so we next tested whether icaritin up-regulated p53 protein expression by repressing Mdm2 expression. As shown in Fig. 4a, icaritin treatment down-regulated Mdm2 protein expression, while CoIP revealed that icaritin reduced the interaction between Mdm2 and p53 (Fig. 4b). Then we examined the effect of Mdm2 protein on inhibtion of p53 ubiquitination caused by icaritin. We introduced exogenous Mdm2 and Ub into HepG2 and SMMC7721 cells, and added icaritin after 28 h. Cells were harvested 14 h after the addition of icaritin following treatment with 10 μM proteasome inhibitor MG132 for 6 h, and ubiquitinated p53 was detected by western blot. As shown in Fig. 4c, icaritin inhibited p53 ubiquitination, while Mdm2 protein overexpression rescued the inhibition in HepG2 and SMMC7721 cells. These results indicate that icaritin stabilized p53 by inhibiting Mdm2-mediated p53 ubiquitination.

### Icaritin inhibited the proliferation and promoted the apoptosis of HepG2/SMMC7721 cells

**Fig. 5:**
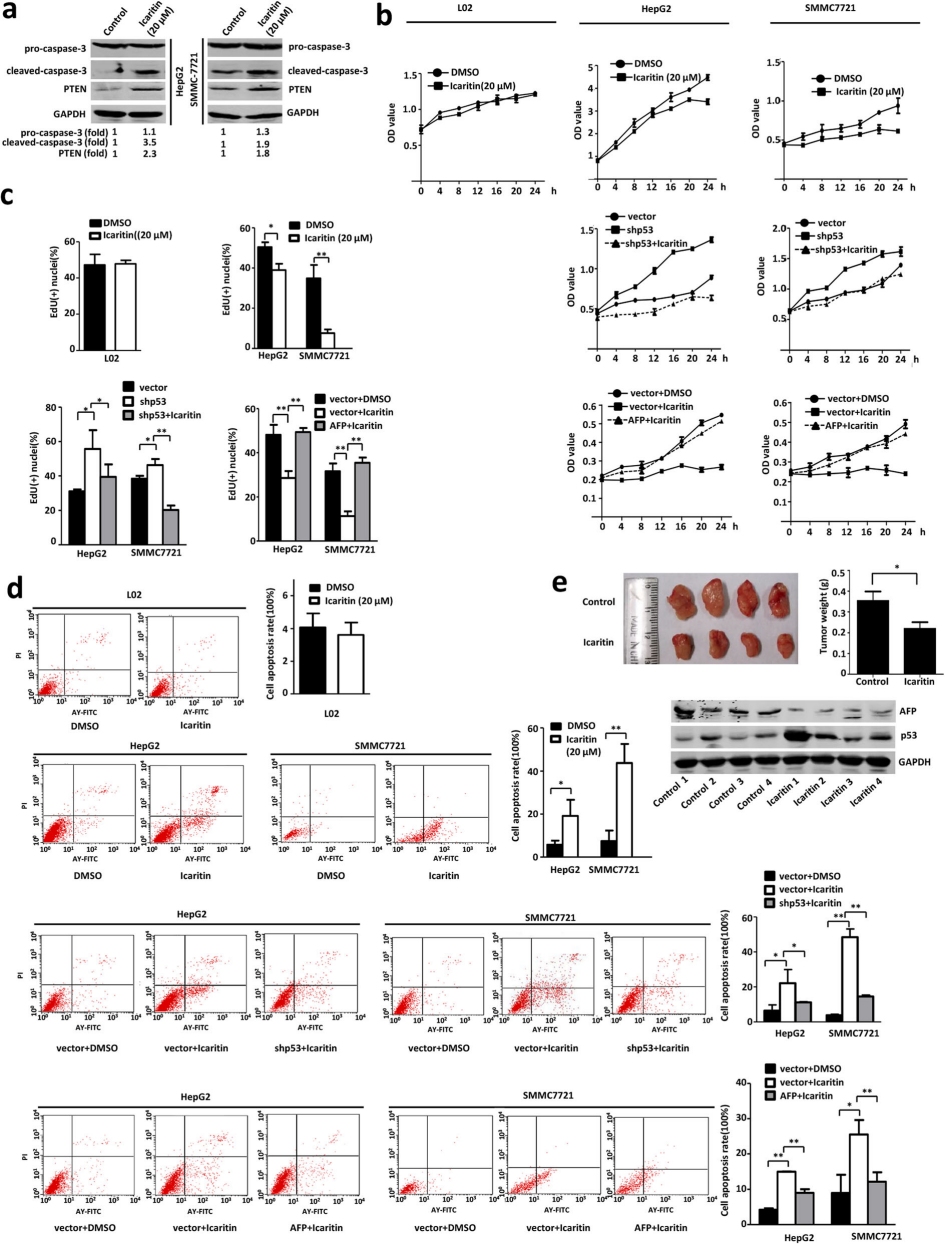
Icaritin inhibited the proliferation and promoted the apoptosis of HepG2/SMMC7721 cells. **a** Icaritin promoted apoptosis and inhibited the proliferation in HCC cells. Pro-caspase-3, cleaved-caspase-3, PTEN and GAPDH (as a control) were determined by western blot in HepG2 and SMMC7721 cells with DMSO or icaritin. **b** P53 knockdown or AFP overexpression attenuated icaritin-induced growth inhibition. The cell growth of icaritin-treated L02 and HepG2 and SMMC7721 cells was analyzed by MTT (top panel). HepG2 and SMMC7721 cells were stably transfected with or without pLL3.7-shp53 or pLL3.7 (as a control) individually (middle panel), or transfected with pcDNA3.1-AFP or pcDNA3.1 (as a control), treated with DMSO or 20 μM icaritin (bottom panel), and then cell growth was analyzed by MTT assay. **c** P53 knockdown or AFP overexpression attenuated icaritin-induced proliferation inhibition. The cell lines from (**b**) were treated with 20 μM icaritin or DMSO and then cell proliferation was analyzed by EdU assay. **d** P53 knockdown or AFP overexpression attenuated icaritin-induced apoptosis. The cell lines from (**b**) were treated with icaritin or DMSO for 20 h and then cell apoptosis was analyzed by flow cytometry. **e** Icaritin inhibited tumor activity in a xenograft mouse model, and the relationship between p53 and AFP in xenograft mouse. HepG2 cells were subcutaneously injected into nude mice. Icaritin was given at a dose of 100 mg/kg via intraperitoneal injection three times a week. The control group was given an equivalent amount of vehicle solvent. Four weeks after injection, the tumors were weighed, and size was measured. Data are shown as mean ± S.D. (*n* = 4). Expression levels of p53, AFP and GAPDH (as a control) in mice tumors were determined by western blot. *, *p* < 0.05; **, *p* < 0.01. The full-length images for blots in Fig. 5**a** and **e** were presented in Supplementary Fig. 8

To further determine the role of the p53/AFP pathway in the effect of icaritin on hepatoma cells, western blot, MTT assays, EdU assays, and flow cytometric analysis were performed. In Fig. 5a, activated caspase 3 and PTEN were up-regulated after icaritin treatment. MTT and EdU assays showed that icaritin decreased the proliferation of HepG2 and SMMC7721 cells compared with control cells treated with DMSO (Fig. 5b and c). However, icaritin had little effect on the proliferation of L02 human liver cells (Fig. 5b and c). These results implied that icaritin has a selective anticancer effect. To investigate the role of p53 protein in icaritin-induced cellular proliferation inhibition, we constructed p53 knockdown HepG2/SMMC7721 cells. As shown in Fig. 5b and c, when p53 was knocked down by shRNA the viability and proliferation of HCC cells was increased; this effect was rescued by icaritin. We also verified that p53 knockdown inhibited icaritin-induced apoptosis (Fig. 5d). Taken together, these data showed that icaritin decreased HCC cell proliferation and promoted HCC cell apoptosis through up-regulating p53 protein expression.

To determine the role of AFP in icaritin-induced cellular proliferation, we constructed AFP-overexpressing HepG2/SMMC7721 cells treated with icaritin. Figure 5b and c showed that icaritin inhibited HCC cell proliferation and AFP overexpression rescued this inhibition. We also used flow cytometry to reveal that icaritin increased the percentage of apoptotic HCC cells compared with the DMSO group and AFP overexpression inhibited the apoptosis induced by icaritin. However, icaritin had little effect on the apoptosis of L02 human liver cells (Fig. 5d). These results suggest that icaritin promoted cell apoptosis by increasing p53 protein expression and down-regulating AFP protein expression. In order to assess the effect of icaritin on the tumorigenic behavior of liver cancer cells in vivo, we tested xenograft growth with HepG2 cells in a nude mouse model. As shown in Fig. 5e, Icaritin inhibited tumorigenesis of HepG2 cells. We extracted tumor tissue protein and performed western blot. The data showed p53 was upregulated and AFP was reduced in icaritin-treated HCC xenografts. According mentioned above, icaritin inhibited cell proliferation and promoted cell apoptosis by regulating the p53–AFP signaling pathway. Compared to icaritin, the effect of standard chemotherapy drug gemcitabine (GEM) on p53-AFP axis was tested. The data showed GEM had no any effect on p53-AFP axis (Fig.S1).

## Discussion

HCC is the most common primary malignancy of the liver and is predominant in China and other Asian countries [25]. Surgical treatment including liver transplantation is the main form of therapy, but this is hindered by poor prognosis and postoperative recurrence. Traditional chemotherapeutic drugs have little effect on HCC, possibly because of its high level of acquired resistance. Although molecular targeted agents have played a role in HCC treatment since the introduction of sorafenib in 2007, other novel anti-HCC agents remain to be explored [26, 27]. Therefore, there is an urgent need for more effective and less toxic alternative drugs in the treatment of liver cancer.

Here, we investigated the potential of icaritin, a compound purified from the medical herb *Epimedium*, as a drug for the treatment of HCC. We found that icaritin inhibited the expression of cytoplasmic AFP in a dose- and time-dependent manner at both the mRNA and protein expression level.

AFP has been reported to play an important role in the development of liver cancer. For example, silencing *AFP* expression induces growth arrest and apoptosis in human Huh 7 liver cancer cells. Furthermore, intracellular AFP promotes cell proliferation and inhibits apoptosis through binding to proteins associated with cell growth or apoptosis [5–8]. AFP has also been reported to promote tumor escape from immune surveillance. These findings showed that AFP may be used as a target for HCC therapy, and our current results demonstrated an antitumor role for icaritin by inhibiting *AFP* expression in the treatment of HCC.

AFP has been studied for many years since the entire *AFP* sequence was cloned in 1983. *AFP* transcription is mainly controlled by its promoter, enhancer, and silencer in the 5′ region [28, 29]. The silencer was reported to show extremely low activity in fetal mouse livers but higher activity in adult mouse livers, suggesting that it is important in inhibiting AFP expression [30]. Previous studies reported the negative regulation of *AFP* expression by p53 protein through direct binding to specific DNA binding sites in mouse liver cancer cells [24].

In the present study, we analyzed the activity of elements in the 5′ regulatory region of *AFP* and the impact of icaritin on its promoter. A p53 binding site was previously identified between − 860 and − 830 bp of the mouse *AFP* promoter [24], and we herein showed for the first time the presence of two p53 binding sites in the 5′ regulatory region of human *AFP* in human liver cancer cells. We also showed that icaritin enhanced the binding of p53 to DNA, and that icaritin treatment led to the down-regulation of *AFP* expression and up-regulation of p53 expression. Moreover, p53 knockdown rescued the icaritin-induced decrease in AFP. These results together suggest that icaritin up-regulated p53 and enhanced the transcriptional inhibition of p53 on AFP protein, thereby reducing AFP expression. At the same time, we detected the expression of histone repressive marker H3K27me3. H3K27me3 was up-regulated after p53 knockdown and inhibited with icaritin treatment (Fig.S2). We considered p53 might also affect AFP expression through mediating repressive via change in chromatin. Cellular p53 levels were previously reported to be mainly controlled by ubiquitin-mediated proteasomal degradation with Mdm2 as the principal endogenous E3 ligase with high specificity for p53 [31]. We also found that the icaritin-induced increase in p53 protein expression was caused by reduced ubiquitination/proteasomal degradation, and that icaritin inhibited Mdm2 expression and the interaction of Mdm2 and p53. These results are consistent with those of previous studies.

HCC is the primary cancer indication considered for icaritin [32]. Earlier studies of icaritin-mediated HCC inhibition mainly focused on signaling pathways involved in proliferation or apoptosis. For example, icaritin was shown to promote the apoptosis of HCC cells by activating the JNK pathway and inducing expression of the Bcl-2 family. Icaritin was also reported to induce the mitochondrial/caspase apoptotic pathway by decreasing the bcl-2/bax protein ratio and activating caspase-3 in SMMC7721 cells [18]. However, the effect of icaritin on p53 ubiquitination has never been reported until now. In our manuscript, we demonstrated icaritin inhibited HCC cellular proliferation and promoted cellular apoptosis through p53/AFP pathway. But more than one target protein is involved in the mechanism of icaritin. We also demonstrated icaritin inhibited AFP gene expression in p53-mutant cell line PLC (Fig.S3). In addition, icaritin was reported to inhibited AFP expression through promoting the expression of miR-1270, miR-1236 and miR-620 in PLC cells [21], MiR-620, miR-1236, miR-1270 might inhibit HCC apoptosis by down-regulating expression of AFP; There are other mechanisms that are independent of p53 and AFP. In p53- mutant HCC cell line Huh 7 and p53-wildtype HCC cell line HepG2, icaritin can inhibit tumor growth by inhibiting activity of SphK. And, icaritin can induce cellular senescence to inhibit HCC cancer in Huh 7 and HepG2 cells [33]. As above mentioned, icaritin plays a wide range of antitumor activity, more detailed mechanisms still need to be further studied.

## Conclusion

**Fig. 6:**
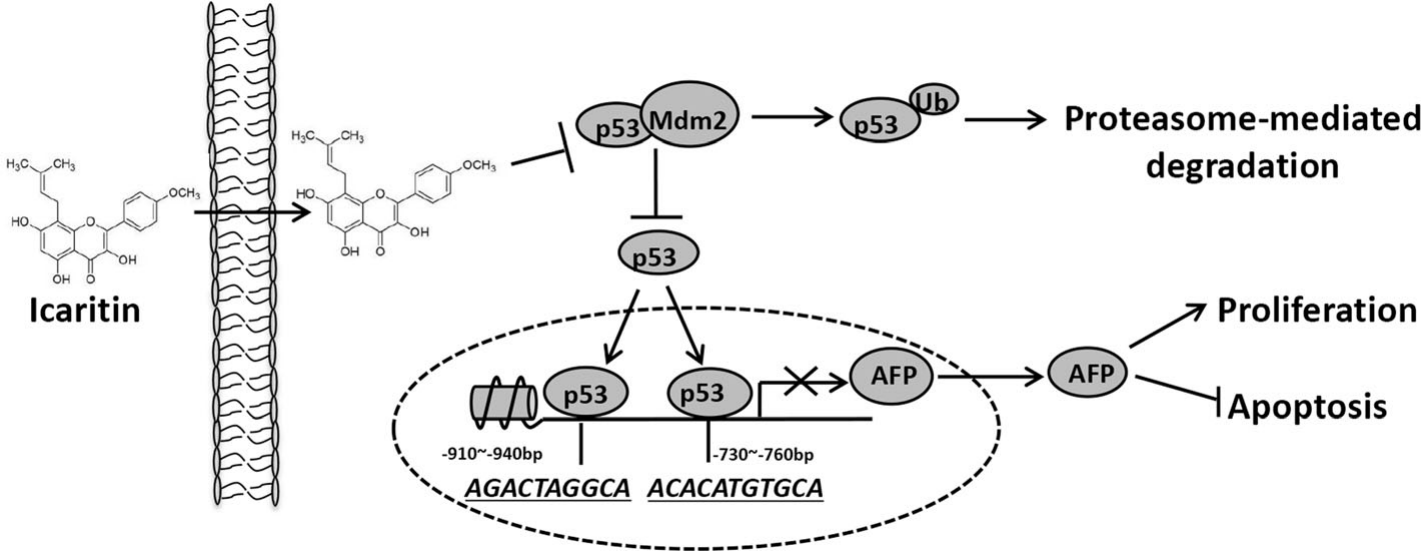
Model for p53 regulating AFP gene expression and its role in icaritin induced HCC inhibition. P53 can negatively regulate AFP expression through direct binding to the promoter of AFP. Icaritin abrogates Mdm2-mediated p53 degradation through down-regulating Mdm2 protein expression and inhibiting the interaction between p53 and Mdm2. The stabilized p53 then inhibits AFP transcription and lead to AFP protein decrease. AFP protein downregulation then inhibits hepatocellular proliferation and promotes hepatocellular apoptosis. Overall, icaritin suppresses cell growth and promotes cell apoptosis through Mdm2/p53–AFP pathway

In conclusion, we showed that icaritin suppressed cell growth and promoted cell apoptosis by inhibiting AFP expression in HCC cells. Icaritin abrogated Mdm2-mediated p53 degradation to stabilize the p53 protein which then inhibited *AFP* transcription leading to a decrease in AFP protein expression. Furthermore, icaritin showed little toxicity in L02 human hepatocytes (Fig. 6). This study elucidated the mechanism of icaritin in promoting apoptosis and inhibiting proliferation of HCC cells, and suggested that it has the potential for use in the clinical treatment of HCC.

## Supplementary Information

**Additional file 1: Supplementary Figure 1.** Gemcitabine do not have any effects on p53/AFP axis. (a) The mRNA expression of AFP was measured by qRT-PCR in HepG2 or SMMC7721 cells treated with GEM. (b) The protein expression of AFP, p53, Mdm2 and GAPDH (as a control) were measured by western blot in HepG2 or SMMC7721 cells treated with Control pbs or 0.5 μM GEM for 24 h. (c) HepG2 cells and SMMC7721 cells were treated with PBS or 0.5 μM GEM respectively. Cells were incubated with the protein translation inhibitor cycloheximide (CHX) for 0, 15, 30, 45, 60, or 120 min before harvest. P53 and GAPDH (as a control) were detected by western blot. (d) EdU assay was used to detect the proliferation of HepG2 and SMMC7721 cells after 0.5 μM GEM treatment for 24 h. (e) The apoptosis of GEM-treated HepG2 and SMMC7721 cells was analyzed by flow cytometry. Results are representative of three independent experiments, and values are the mean ± S.E. The full-length images for blots in Fig. S1b and c were presented in Supplementary Fig. 9.

**Additional file 2: Supplementary Figure 2.** P53 knockdown promoted H3K27me3 expression and icaritin inhibited H3K27me3 expression. Western blot was performed to detect the expression of p53, H3K27me3 and GAPDH (as a control) in HepG2 and SMMC7721 cells with p53 knockdown or icaritin treatment. The full-length images for blots in Fig. S2 were presented in Supplementary Fig. 10.

**Additional file 3: Supplementary Figure 3.** Icaritin inhibited AFP expression in PLC cells. Western blot was used to detect the expression of AFP and β-actin (as a control) in PLC cells with Control DMSO or icaritin. The full-length images for blots in Fig. S3 were presented in Supplementary Fig. 11.

**Additional file 4: Supplementary Figure 4.** The full-length gel images of western blots in Fig. 1a.

**Additional file 5: Supplementary Figure 5.** The full-length gel images of gels and western blots in Fig. 2b and d.

**Additional file 6: Supplementary Figure 6.** The full-length gel images of western blots in Fig. 3b and c.

**Additional file 7: Supplementary Figure 7.** The full-length gel images of western blots in Fig. 4a, b and c.

**Additional file 8: Supplementary Figure 8.** The full-length gel images of western blots in Fig. 5a and e.

**Additional file 9: Supplementary Figure 9.** The full-length gel images of western blots in Supplementary Fig. 1b and c.

**Additional file 10: Supplementary Figure 10.** The full-length gel images of western blots in Supplementary Fig. 2.

**Additional file 11: Supplementary Figure 11.** The full-length gel images of western blots in Supplementary Fig. 3.

## Data Availability

The datasets used and/or analyzed during the current study are available from the corresponding author on reasonable request.

## Abbreviations

AFP: Alpha fetoprotein
RA: Retinoic acid
RAR: Retinoic acid receptors
GADD153: Growth arrest and DNA damage-inducible 153 gene
GADD45A: Growth arrest and DNA damage- inducible 45α gene
Fn14: Fibroblast growth factor-inducible 14
FBS: Fetal bovine serum
DAPI: 2-(4-amidinophenyl)-6-Indolecarbamidine
MTT: 3-(4,5-dimethylthiazol-2-yl) -2,5 diphenyltetrazolium bromide
EdU: 5-Ethynyl-2-deoxyuridine
Mdm2: Mouse double minute protein 2
