# Supplementary figures and images for: Icaritin promotes apoptosis and inhibits proliferation by down-regulating AFP gene expression in hepatocellular carcinoma
Source: BMC Cancer. 2021 Mar 25;21:318. doi: 10.1186/s12885-021-08043-9 (PMC7992931; doi:10.1186/s12885-021-08043-9)

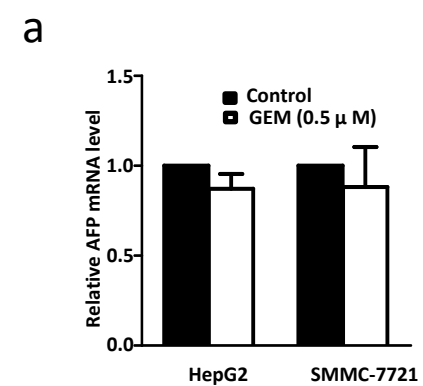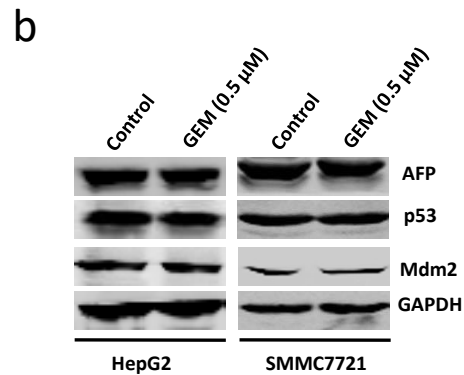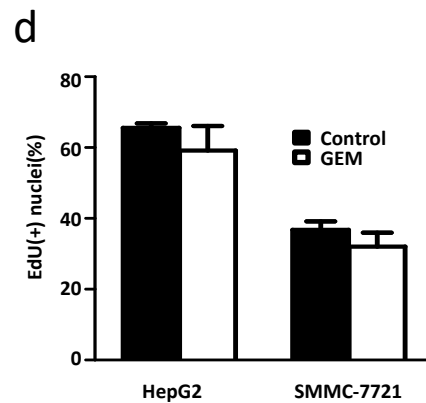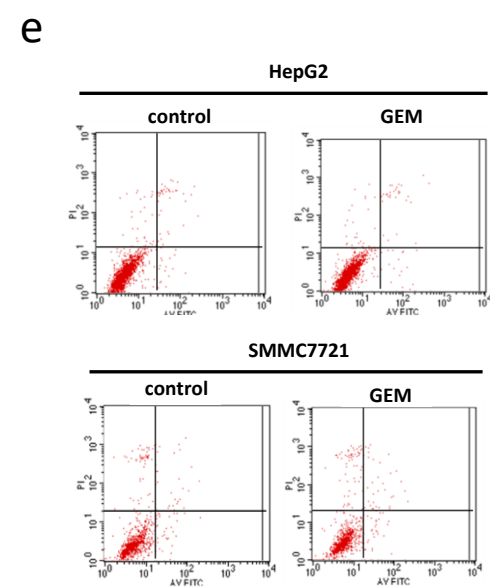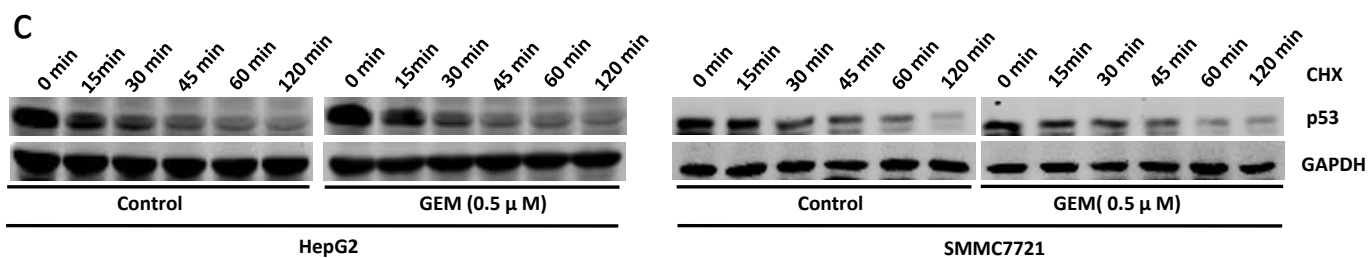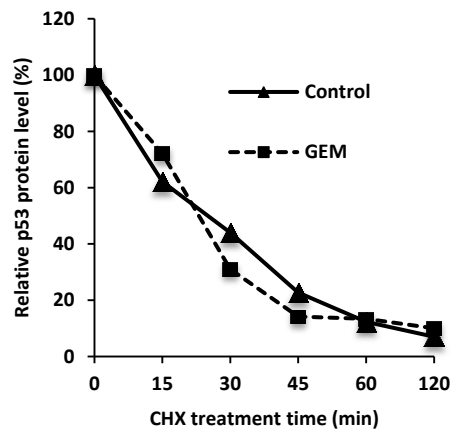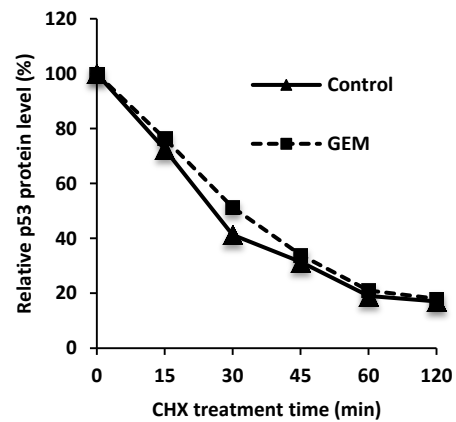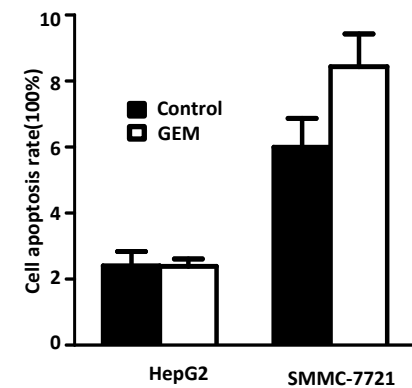

Supplement: Supplementary file 1 — Additional file 1: Supplementary Figure 1. Gemcitabine do not have any effects on p53/AFP axis. (a) The mRNA expression of AFP was measured by qRT-PCR in HepG2 or SMMC7721 cells treated with GEM. (b) The protein expression of AFP, p53, Mdm2 and GAPDH (as a control) were measured by western blot in HepG2 or SMMC7721 cells treated with Control pbs or 0.5 μM GEM for 24 h. (c) HepG2 cells and SMMC7721 cells were treated with PBS or 0.5 μM GEM respectively. Cells were incubated with the protein translation inhibitor cycloheximide (CHX) for 0, 15, 30, 45, 60, or 120 min before harvest. P53 and GAPDH (as a control) were detected by western blot. (d) EdU assay was used to detect the proliferation of HepG2 and SMMC7721 cells after 0.5 μM GEM treatment for 24 h. (e) The apoptosis of GEM-treated HepG2 and SMMC7721 cells was analyzed by flow cytometry. Results are representative of three independent experiments, and values are the mean ± S.E. The full-length images for blots in Fig. S1b and c were presented in Supplementary Fig. 9. [file 12885_2021_8043_MOESM1_ESM.pdf]

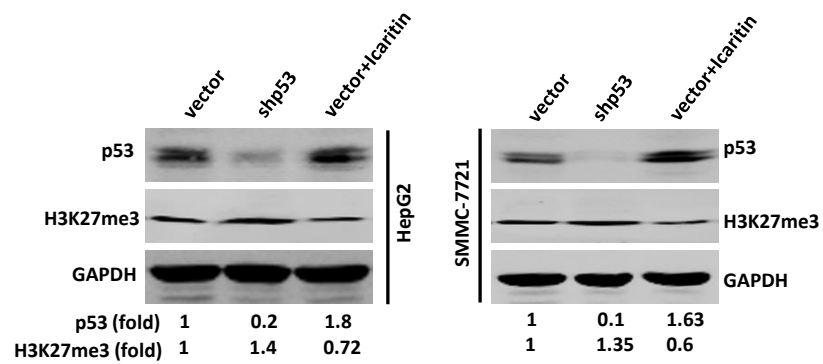

Supplement: Supplementary file 2 — Additional file 2: Supplementary Figure 2. P53 knockdown promoted H3K27me3 expression and icaritin inhibited H3K27me3 expression. Western blot was performed to detect the expression of p53, H3K27me3 and GAPDH (as a control) in HepG2 and SMMC7721 cells with p53 knockdown or icaritin treatment. The full-length images for blots in Fig. S2 were presented in Supplementary Fig. 10. [file 12885_2021_8043_MOESM2_ESM.pdf]

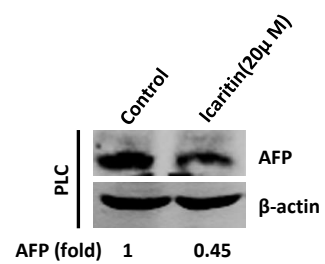

Supplement: Supplementary file 3 — Additional file 3: Supplementary Figure 3. Icaritin inhibited AFP expression in PLC cells. Western blot was used to detect the expression of AFP and β-actin (as a control) in PLC cells with Control DMSO or icaritin. The full-length images for blots in Fig. S3 were presented in Supplementary Fig. 11. [file 12885_2021_8043_MOESM3_ESM.pdf]

Original gels and blots of AFP and β-actin in PLC cells (Corresponding to Fig. S3 in the manuscript).


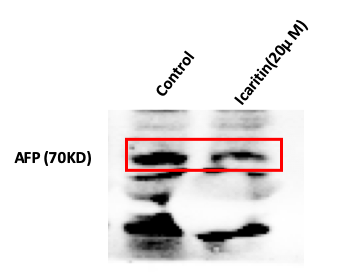


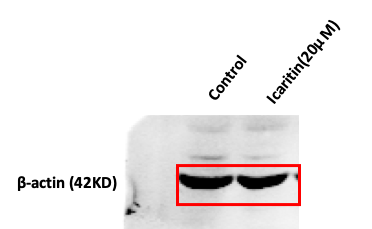

Supplement: Supplementary file 11 — Additional file 11: Supplementary Figure 11. The full-length gel images of western blots in Supplementary Fig. 3. [file 12885_2021_8043_MOESM11_ESM.docx]
