# Supplementary material for: Icaritin promotes apoptosis and inhibits proliferation by down-regulating AFP gene expression in hepatocellular carcinoma
Source: BMC Cancer. 2021 Mar 25;21:318. doi: 10.1186/s12885-021-08043-9 (PMC7992931; doi:10.1186/s12885-021-08043-9)

Original gels and blots of AFP and GAPDH in HepG2 cells and SMMC7721 cells with icaritin treatment at different concentrations. (Corresponding to Fig. 1a in the manuscript).


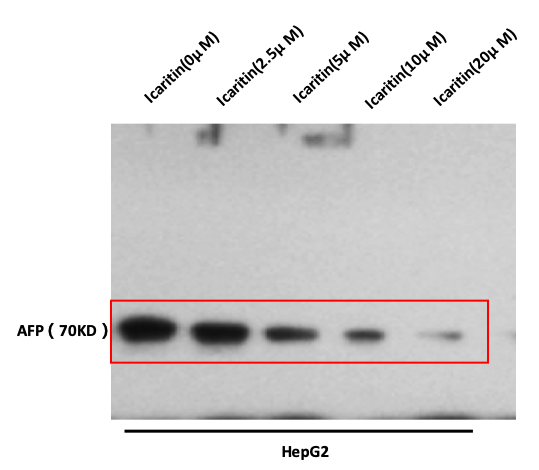


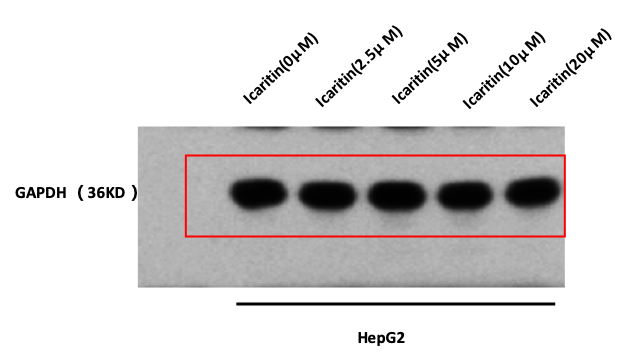


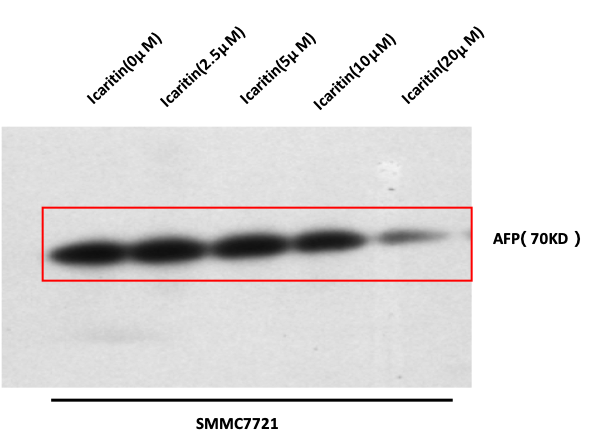


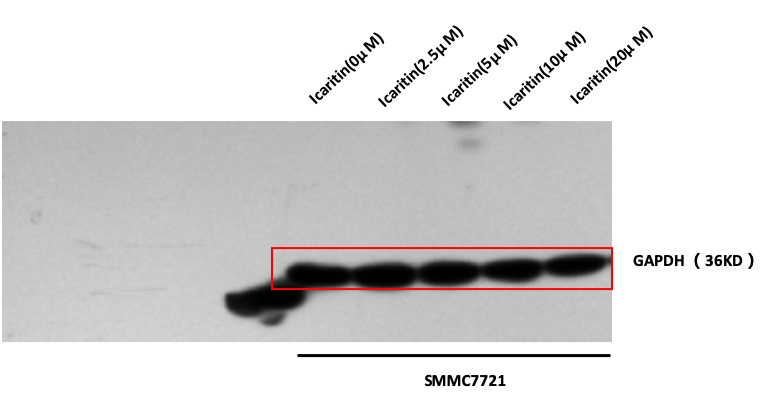

Supplement: Supplementary file 4 — Additional file 4: Supplementary Figure 4. The full-length gel images of western blots in Fig. 1a. [file 12885_2021_8043_MOESM4_ESM.docx]
