# Supplementary material for: Icaritin promotes apoptosis and inhibits proliferation by down-regulating AFP gene expression in hepatocellular carcinoma
Source: BMC Cancer. 2021 Mar 25;21:318. doi: 10.1186/s12885-021-08043-9 (PMC7992931; doi:10.1186/s12885-021-08043-9)

Original gels and blots of specific PCR for detecting p53 affinity in AFP 5’ promoter region and the effect of icaritin in HepG2 cells and SMMC7721 cells (Corresponding to Fig. 2b in the manuscript).


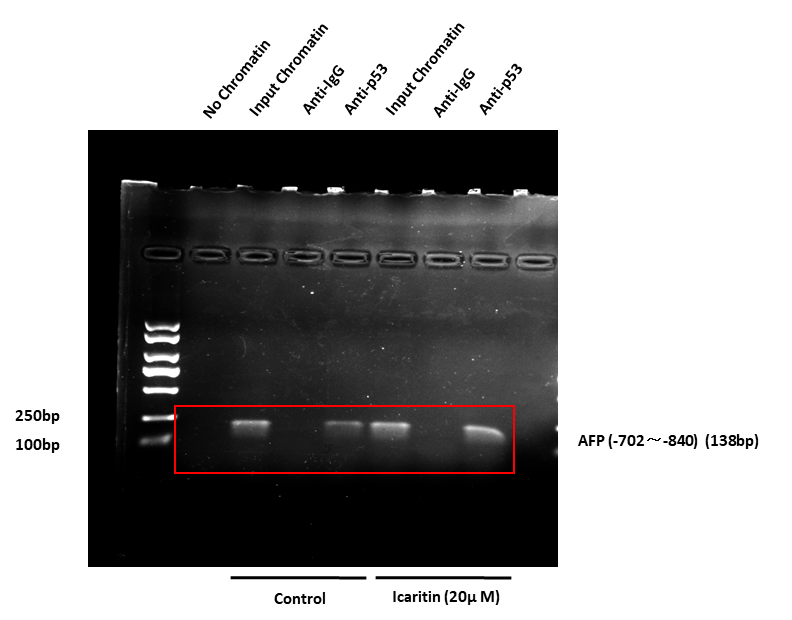


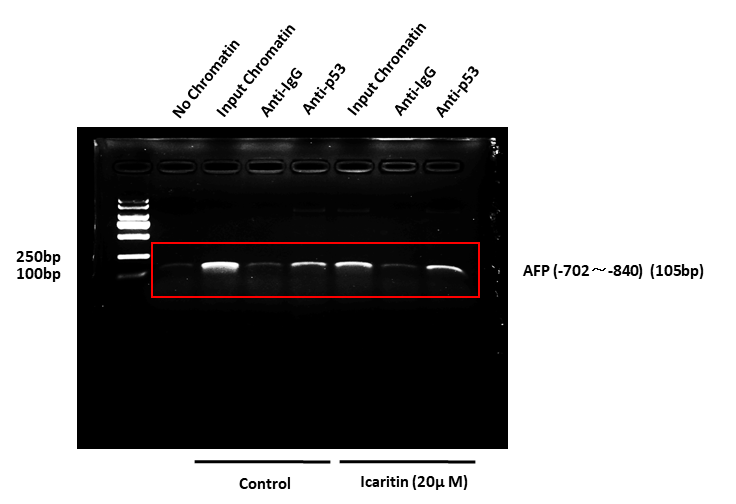


Original gels and blots of AFP, p53, p-p53, ARF^p14^ and GAPDH in HepG2 cells and SMMC7721 cells (Corresponding to Fig. 2d in the manuscript).


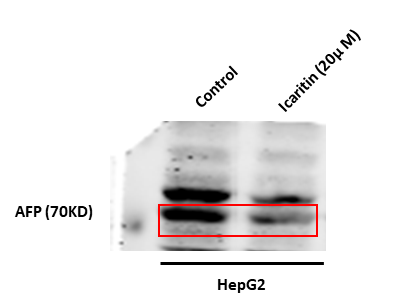

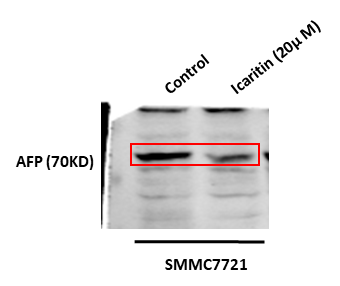


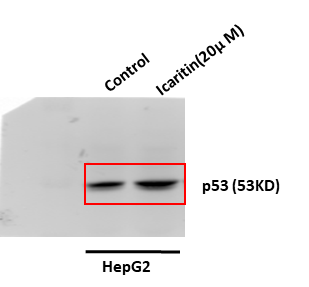

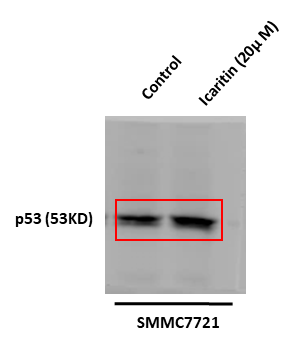


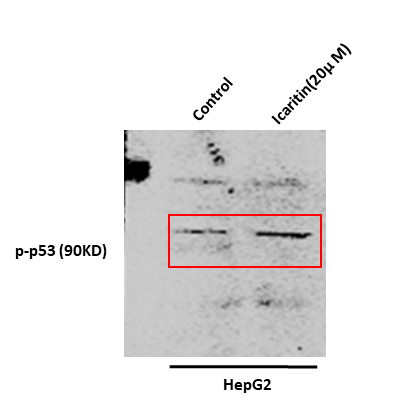

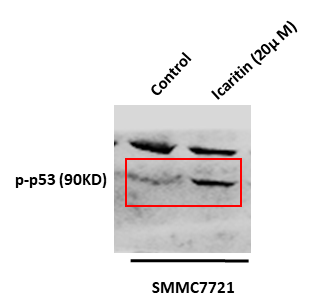


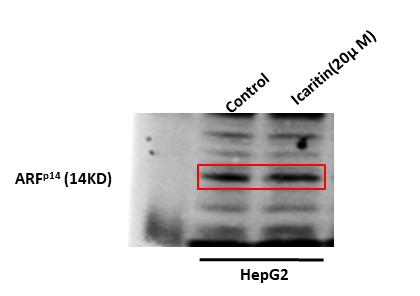

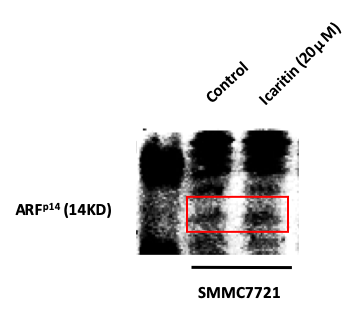


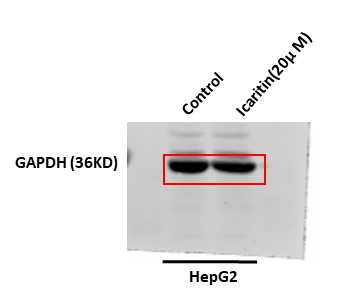

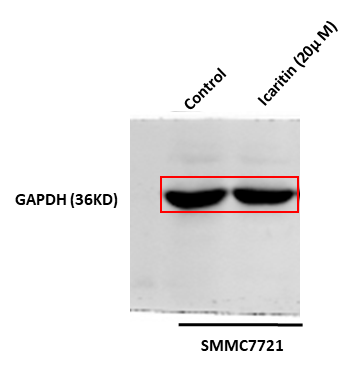


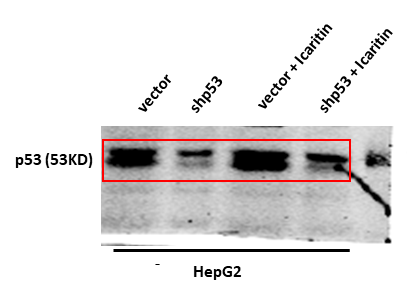


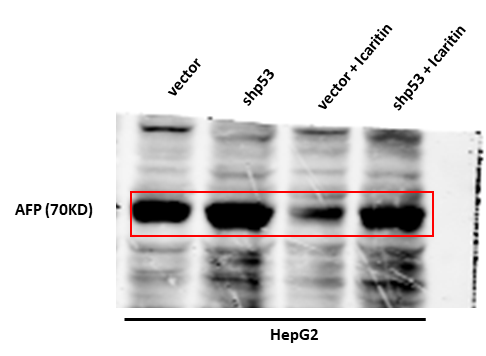


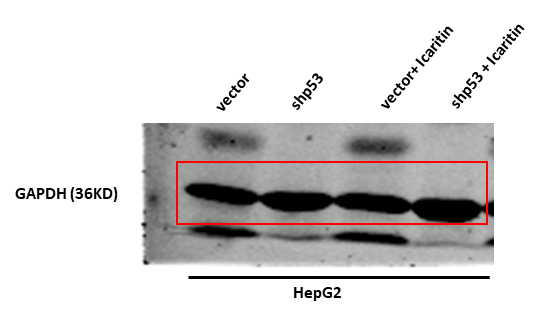


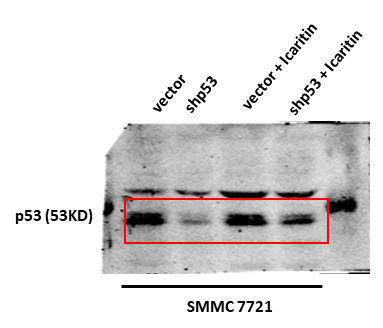


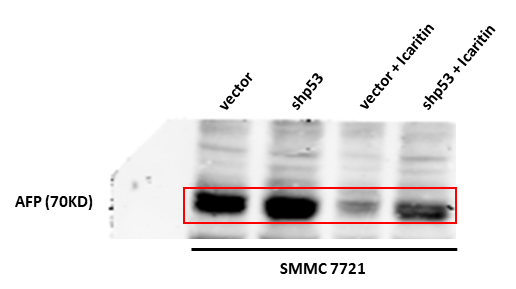


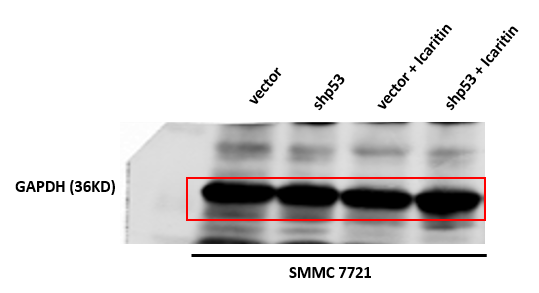

Supplement: Supplementary file 5 — Additional file 5: Supplementary Figure 5. The full-length gel images of gels and western blots in Fig. 2b and d. [file 12885_2021_8043_MOESM5_ESM.docx]
