# Supplementary material for: Icaritin promotes apoptosis and inhibits proliferation by down-regulating AFP gene expression in hepatocellular carcinoma
Source: BMC Cancer. 2021 Mar 25;21:318. doi: 10.1186/s12885-021-08043-9 (PMC7992931; doi:10.1186/s12885-021-08043-9)

Original gels and blots of p53 and GAPDH in HepG2 cells and SMMC7721 cells (Corresponding to Fig. 3b in the manuscript).


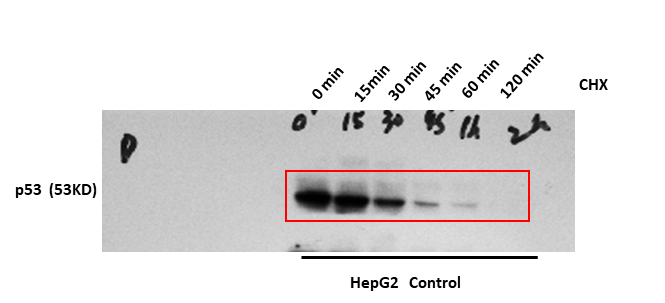


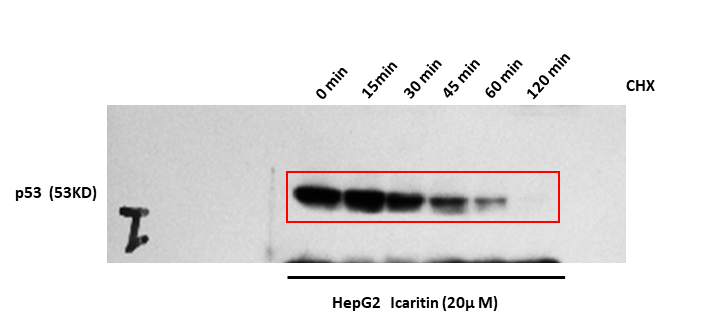


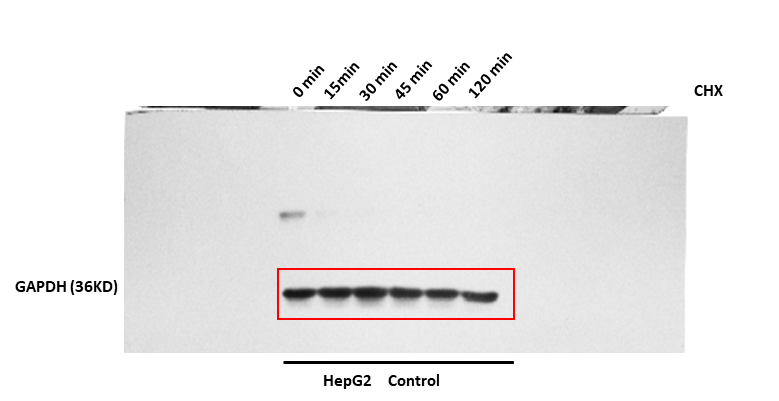


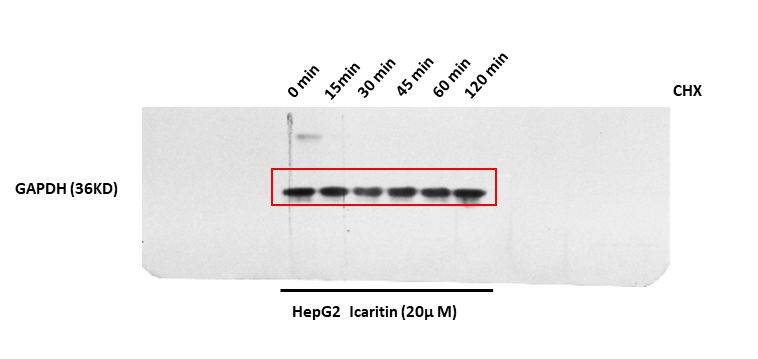


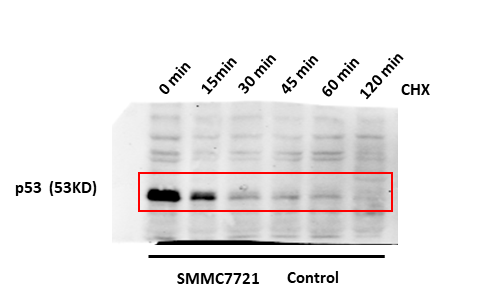


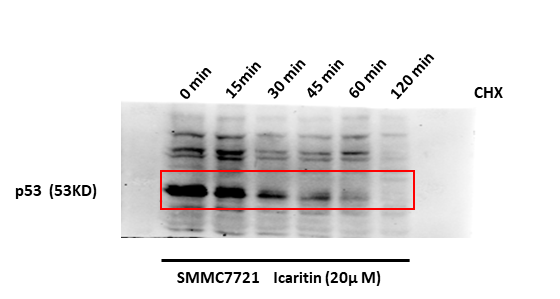


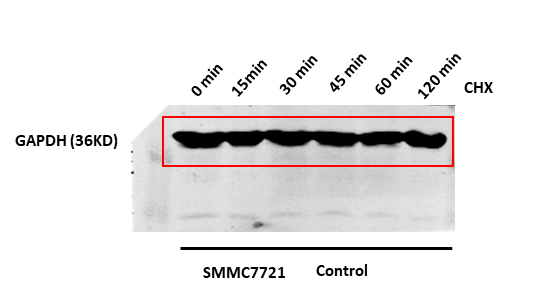


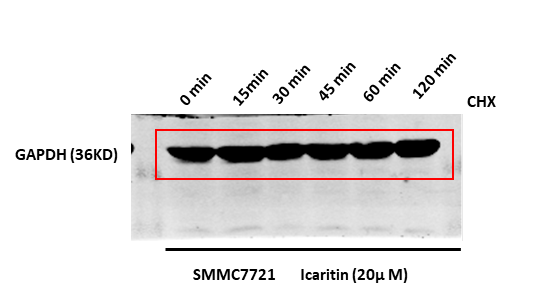


Original gels and blots of Ub-p53 and p53 (Corresponding to Fig. 3c in the manuscript).


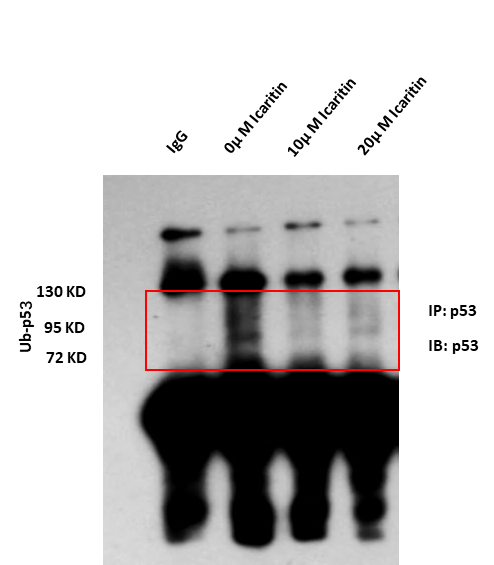


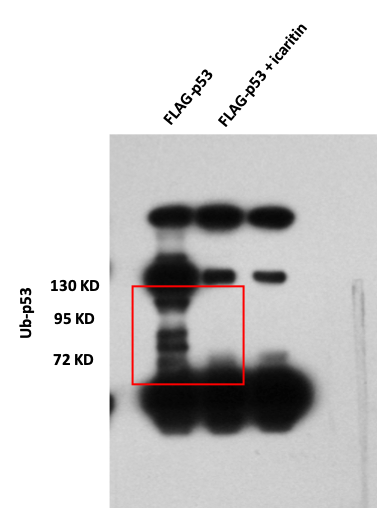


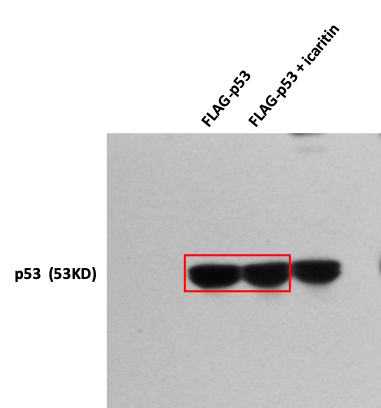

Supplement: Supplementary file 6 — Additional file 6: Supplementary Figure 6. The full-length gel images of western blots in Fig. 3b and c. [file 12885_2021_8043_MOESM6_ESM.docx]
