# Supplementary material for: Icaritin promotes apoptosis and inhibits proliferation by down-regulating AFP gene expression in hepatocellular carcinoma
Source: BMC Cancer. 2021 Mar 25;21:318. doi: 10.1186/s12885-021-08043-9 (PMC7992931; doi:10.1186/s12885-021-08043-9)

Original gels and blots of AFP, p53, Mdm2 and GAPDH in HepG2 cells and SMMC7721 cells (Corresponding to Fig. S1b in the manuscript).


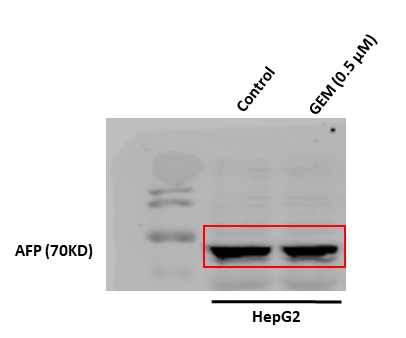


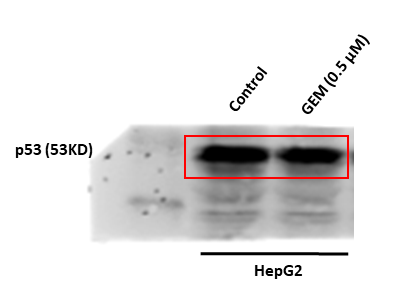


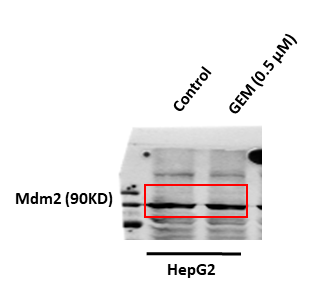


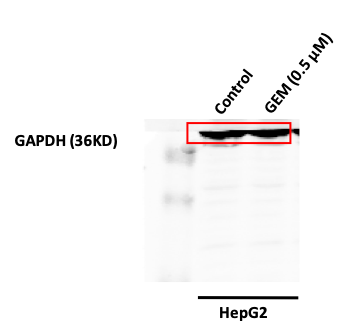


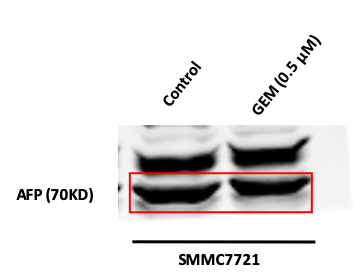


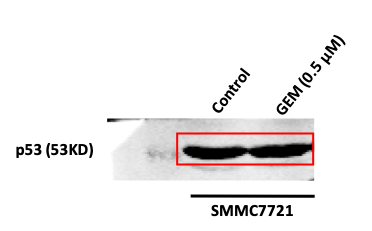


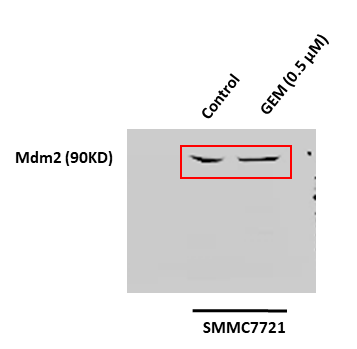


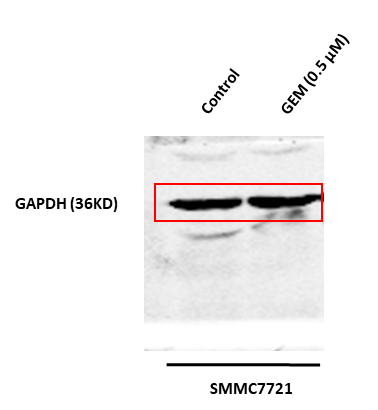


Original gels and blots of p53 and GAPDH in HepG2 cells and SMMC7721 cells (Corresponding to Fig. S1c in the manuscript).


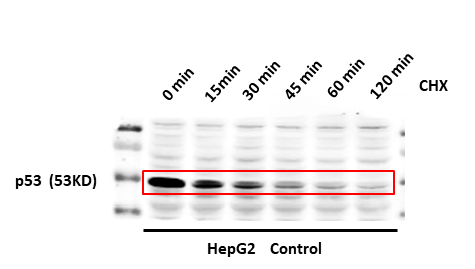


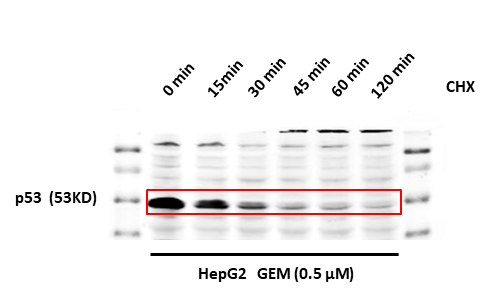


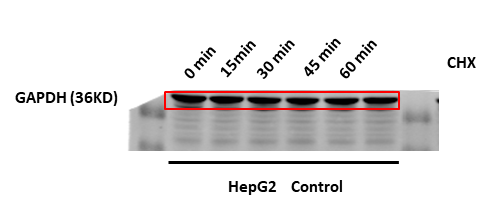


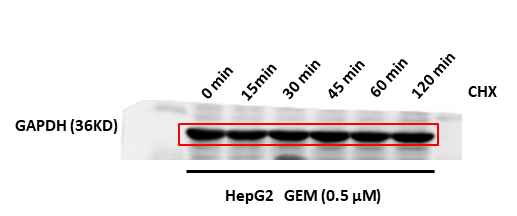


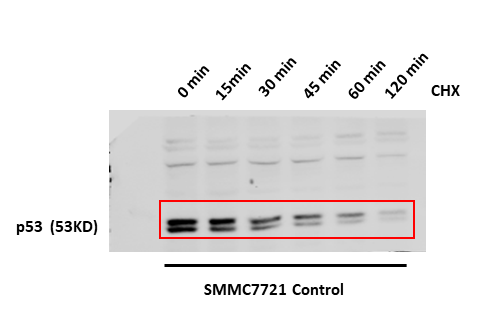


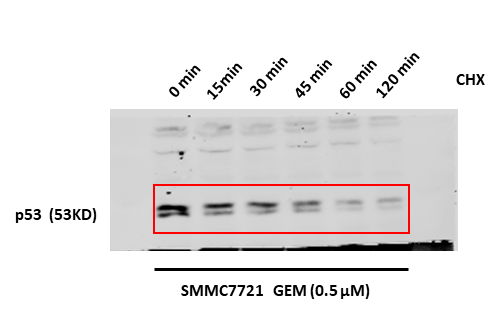


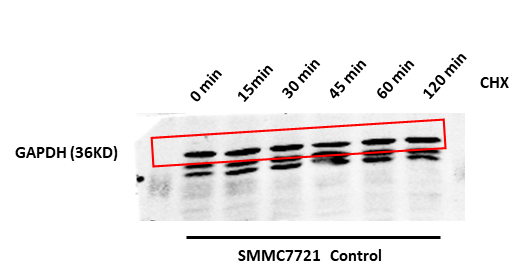


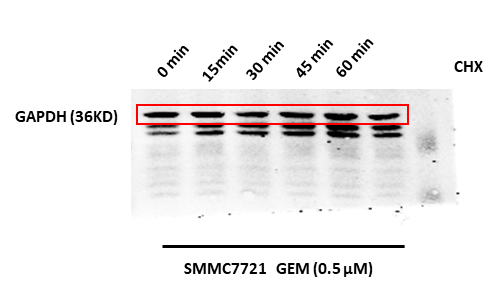

Supplement: Supplementary file 9 — Additional file 9: Supplementary Figure 9. The full-length gel images of western blots in Supplementary Fig. 1b and c. [file 12885_2021_8043_MOESM9_ESM.docx]
