# Supplementary material for: Icaritin promotes apoptosis and inhibits proliferation by down-regulating AFP gene expression in hepatocellular carcinoma
Source: BMC Cancer. 2021 Mar 25;21:318. doi: 10.1186/s12885-021-08043-9 (PMC7992931; doi:10.1186/s12885-021-08043-9)

Original gels and blots of p53, H3k27me3 and GAPDH in HepG2 cells and SMMC7721 cells (Corresponding to Fig. S2 in the manuscript).


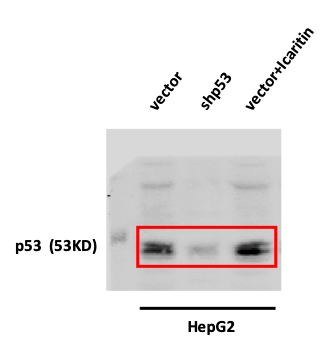

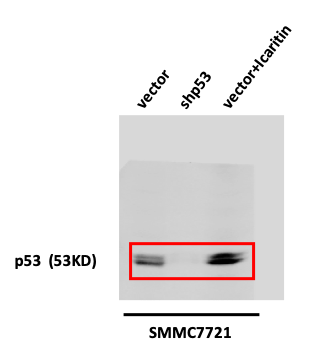

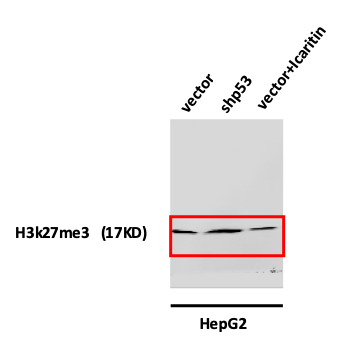

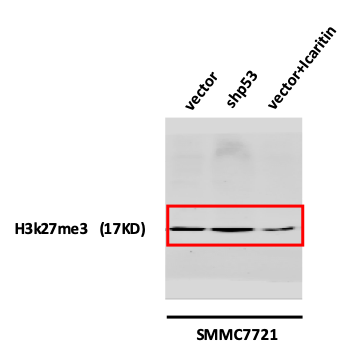


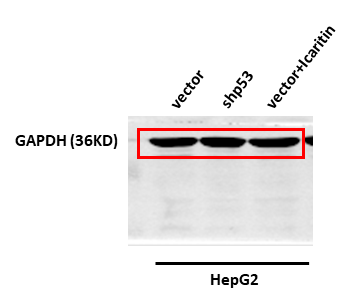

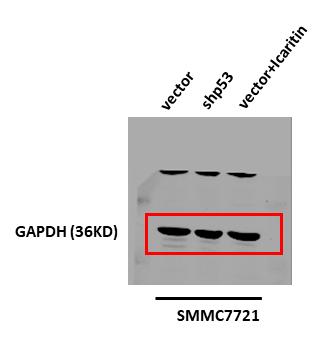

Supplement: Supplementary file 10 — Additional file 10: Supplementary Figure 10. The full-length gel images of western blots in Supplementary Fig. 2. [file 12885_2021_8043_MOESM10_ESM.docx]
